# EphB2 receptor tyrosine kinase promotes hepatic fibrogenesis in mice via activation of hepatic stellate cells

**DOI:** 10.1038/s41598-018-20926-9

**Published:** 2018-02-07

**Authors:** Patrice N. Mimche, Choon M. Lee, Sylvie M. Mimche, Manoj Thapa, Arash Grakoui, Mark Henkemeyer, Tracey J. Lamb

**Affiliations:** 1 Division of Microbiology and Immunology, Department of Pathology, University of Utah School of Medicine, Salt Lake City, UT USA; 2 Department of Pharmacology, Emory University School of Medicine, Atlanta, GA USA; 3 Division of Medicine, Department of Infectious Diseases, Emory Vaccine Center, Emory University School of Medicine, Atlanta, GA USA; 4 Department of Neuroscience, University of Texas Southwestern Medical Center, Dallas, TX USA

## Abstract

Hepatic fibrosis is the result of an excessive wound-healing response subsequent to chronic liver injury. A feature of liver fibrogenesis is the secretion and deposition of extracellular matrix proteins by activated hepatic stellate cells (HSCs). Here we report that upregulation of EphB2 is a prominent feature of two mouse models of hepatic fibrosis and also observed in humans with liver cirrhosis. EphB2 is upregulated and activated in mouse HSCs following chronic carbon tetrachloride (CCl_4_) exposure. Moreover, we show that EphB2 deficiency attenuates liver fibrosis and inflammation and this is correlated with an overall reduction in pro-fibrotic markers, inflammatory chemokines and cytokines. In an *in vitro* system of HSCs activation we observed an impaired proliferation and sub-optimal differentiation into fibrogenic myofibroblasts of HSCs isolated from *EphB2−/*− mice compared to HSCs isolated from wild type mice. This supports the hypothesis that EphB2 promotes liver fibrosis partly via activation of HSCs. Cellular apoptosis which is generally observed during the regression of liver fibrogenesis was increased in liver specimens of CCl_4_-treated *EphB2−/*− mice compared to littermate controls. This data is suggestive of an active repair/regeneration system in the absence of EphB2. Altogether, our data validate this novel pro-fibrotic function of EphB2 receptor tyrosine kinase.

## Introduction

Hepatic fibrosis is the result of an overwhelming wound-healing process subsequent to chronic liver injury. Cirrhosis is the end-stage of liver fibrosis and is the leading cause of mortality associated with liver pathologies^1,2^. Despite progress made in the understanding of the molecular mechanisms underlying the progression of hepatic fibrogenesis, FDA-approved treatments are still severely limited. A prominent feature of liver fibrosis is the excessive deposition of extracellular matrix proteins by activated hepatic stellate cells (HSCs) which have been differentiated into fibrogenic myofibroblasts^3–5^. This activation process is further driven by inflammation and recruitment of immune cells to the site of liver injury, therefore providing the pro-inflammatory/fibrogenic microenvironment critical for the transdifferentiation of quiescent HSCs into fibrogenic myofibroblasts^6^. However, the effector molecules that potentiate the generation of fibrogenic myofibroblasts at the onset and progression of fibrosis have not been fully elucidated. The development of novel therapies to treat the life-threatening complications of liver fibrosis will require the identification of the molecules that drive this process.

The Eph (Erythropoietin producing hepatocellular) receptor tyrosine kinases and their corresponding ephrin ligands (Eph receptor interacting) are cell-bound molecules expressed in various tissues^7^. In human and mouse, Eph receptors consist of 14 structurally related members divided into two sub-families: nine Eph-A and five Eph-B receptors that bind respectively to a glycosyl-phosphatidylinositol-linked Ephrin-As (five members) and the transmembrane Ephrin-Bs (three members) cell surface ligands^7^. Originally identified in hepatocellular carcinoma cell line^8^, Eph receptors form the largest family of receptor tyrosine kinases and expression of the Eph/Ephrin system has been described in a variety of cells and tissues in human^9^. Ligation of cell–bound Eph receptors to membrane-tethered ephrin ligands initiates a bi-directional signaling cascade between the receptor expressing (forward signaling) and the ligand-expressing (reverse signaling) cells^10^. Activation of the Eph/Ephrin dependent signaling pathway regulates multiple biological processes including cancer progression^11^, cell migration, proliferation^9,12^, epithelial-to-mesenchymal-transition (EMT)^13,14^, angiogenesis, inflammation and tissue remodelling during embryonic organ development^15^.

To date, a smattering of studies have highlighted key functional properties of the Eph/Ephrin molecules that are also relevant to liver fibrosis^1^. Interaction between EphB4 and Ephrin B2 has been found to modify recruitment of liver sinusoidal endothelial cells and VEGF production, both critical events that are required for the development of hepatic fibrosis^16^. Ephrin-B2 ligand has also been shown to influence the formation and function of hepatic vascular structure by modulating Platelet derived growth factor receptor-β (PDGFR-β) signaling^17^. EphB2 is the most upregulated of the EphB/EphrinB molecules in hepatocellular carcinoma, the end-stage of liver fibrosis/cirrhosis^18^ possibly linking upregulation of EphB2 with disease progression.

EphB2 is an axon guidance cue that was originally identified as a critical mediator of axonogenesis during the early stage of the CNS development^19,20^; emphasizing the link between developmentally regulated pathways and fibrogenesis^1^. Indeed, EphB2 has also been found to be upregulated in fibroproliferative membrane in ocular diseases^21^, psoriatic epidermis^22^, skin wounding^23^, idiopathic pulmonary fibrosis^24^ and in fibrotic scar following CNS injury^25^. We previously showed that EphB2 receptor was upregulated in the livers of mice with malaria-causing *Plasmodium* infection and was implicated in mouse malaria-associated hepatic fibrosis^26^. However, it remains to be determined whether EphB2 could also be involved in non-pathogen driven liver inflammation and fibrosis.

In this report, we provide substantial evidence linking upregulation of EphB2 with liver fibrogenesis in non-pathogen driven mouse models of hepatic fibrosis. We demonstrate that EphB2 is critical for the optimal trans-differentiation of quiescent HSCs into fibrogenic myofibroblasts and accordingly EphB2 deficiency attenuates hepatic fibrosis in mice. Collectively our data confirm the pro-fibrogenic function of EphB2 receptor that could be potentially targeted for anti-fibrotic therapies.

## Results

### EphB2 is upregulated in the liver after fibrotic injury

Previously, we reported that EphB2 is upregulated in fibrotic livers of mice infected with blood stage *Plasmodium* parasites^26^. To ascertain whether this observation is also relevant during liver fibrosis in non-infectious settings in mice, we determined the mRNA expression profile of *EphB* receptors and *Ephrin-B* ligands in two mouse models of hepatic fibrosis: chronic administration of the hepatotoxic chemical carbon tetrachloride (CCl_4_) and the *MDR2*-null mice fed a normal rodent laboratory diet to induce biliary fibrosis^27^.

**Figure 1:**
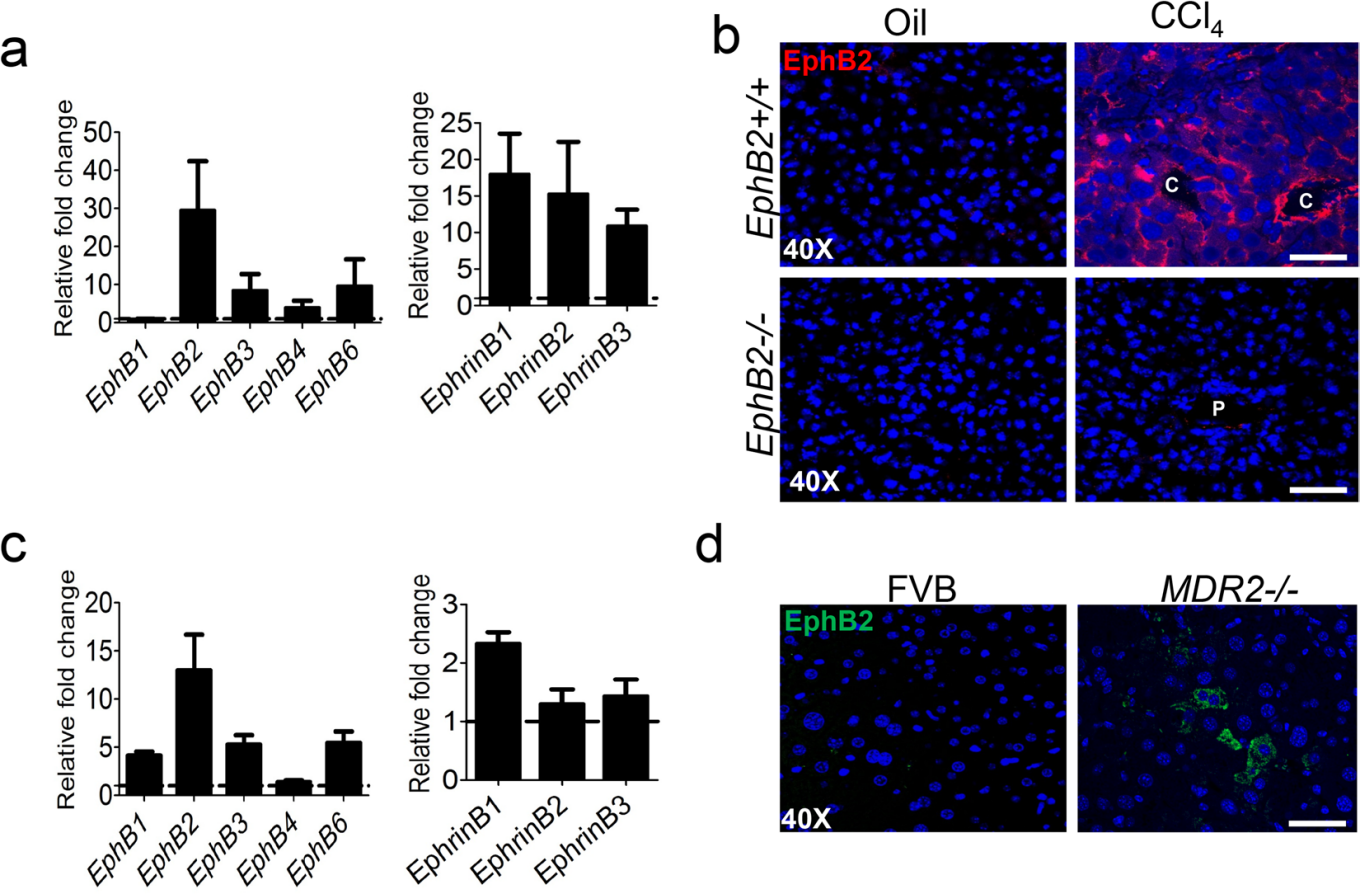
Expression of EphB2 increases in the liver after chronic fibrogenic injury. (**a**) *EphB* receptors and *Ephrin-B* ligands mRNA levels were analyzed in livers of mice subjected to chronic injections of CCl_4_ or vehicle (oil) controls using RT-qPCR. Results are shown as fold change compared to vehicle-treated controls. Error bars represent mean ± SEM. n = 10 animals and are representative of two repeat experiments. (**b**) OCT frozen liver sections of mice subjected to chronic injections of CCl_4_ or vehicle (oil) controls were analyzed for expression of EphB2 protein by immunofluorescence confocal microscopy. Scale bar = 100 µm; “C” denotes the central vein and “P” denotes the portal vein. All images are representative of 5 mice per group. (**c**) *EphB* receptors and *Ephrin-B* ligands mRNA levels were analyzed in livers of *MDR2*-null mice aged 25 weeks and fed a normal diet or FVB control mice as in **a**. Error bars represent mean ± SEM. n = 3 animals. (**d**) FFPE liver sections from MDR2-null mice and their representative controls were analyzed for expression of EphB2 protein using immunofluorescence confocal microscopy. Scale bar = 100 µm; “P” denotes the portal vein. All images are representative of 3 mice per group.

Using real-time quantitative PCR (RT-qPCR), we found that the mRNA levels of *Eph-B2, B3, B4* and *B6*, but not *EphB1* receptors were upregulated in liver tissues of CCl_4_-treated mice compared to oil-treated controls mice (Fig. 1a). *EphB2* was transcriptionally the most upregulated of the *EphB* receptors (*EphB2*~30-fold compared to *EphB3*~8-fold, *EphB4*~4-fold and *EphB6*~10-fold) (Fig. 1a). RNA transcripts of *Ephrin-B* ligands were also elevated in the livers of CCl_4_-treated mice relative to oil-treated controls mice (*EphrinB1*~17-fold, *EphrinB2*~15-fold and *EphrinB3*~10-fold) (Fig. 1a). Immunofluorescence staining of liver sections confirmed an increase in EphB2 protein in CCl_4_-treated mice compared to oil-treated control mice and the specificity of EphB2 antibody used was further validated by an absence of staining in liver sections of *EphB2*−*/*− mice (Fig. 1b).

To ascertain whether EphB2 upregulation could also be observed during acute CCl_4_-mediated liver injury, we screened for *EphB2* mRNA levels after administration of one, three and six doses of CCl_4_. Our data show that *EphB2* mRNA levels increase specifically after repetitive administration of at least 6 doses of CCl_4_ (Supplementary Fig. S1A). The latter findings clearly indicate that *EphB2* transcripts upregulation is mainly observed during chronic CCl_4_ intoxication and this is mirrored by transcription of fibrogenic markers collagen type 1-α-1 chain (*COL1α1*) and α-smooth muscle actin (*αSMA*), (Supplementary Fig. S1B,C).

In the *MDR2*-null mouse model of biliary fibrosis, we also found that the mRNA levels of *Eph-B1, B2, B3 and B6*, but not *EphB4* receptors were upregulated in the livers of *MDR2*-null mice at week 25 compared to control mice (Fig. 1c). Interestingly, *EphB2* mRNA transcripts were again prominent (*EphB2*~13-fold compared to *EphB1*~4-fold, *EphB3*~5-fold and *EphB6*~6-fold) (Fig. 1c) and this was replicated by an immunofluorescence analysis depicting an upregulation of EphB2 protein level in liver sections of *MDR2*-null mice compared to control mice (Fig. 1d). However, transcripts for *Ephrin-B* ligands were marginally upregulated in *MDR2*-null mice; ≤2-fold for each gene (Fig. 1c).

Having established that EphB2 is the most highly upregulated of the EphB receptors in both the CCl_4_ and *MDR2*-null mouse models of hepatic fibrosis, we next determined the human relevance of our findings by screening for EphB2 expression in human biopsies specimen of cirrhotic livers. In most cases EphB2 proteins were elevated in liver cirrhosis but virtually undetected in normal liver (Supplementary Fig. S2). Altogether, this data demonstrates that upregulation of EphB2 is a feature of mouse and human liver fibrosis.

### EphB2 is expressed and activated on HSCs in fibrotic liver

**Figure 2:**
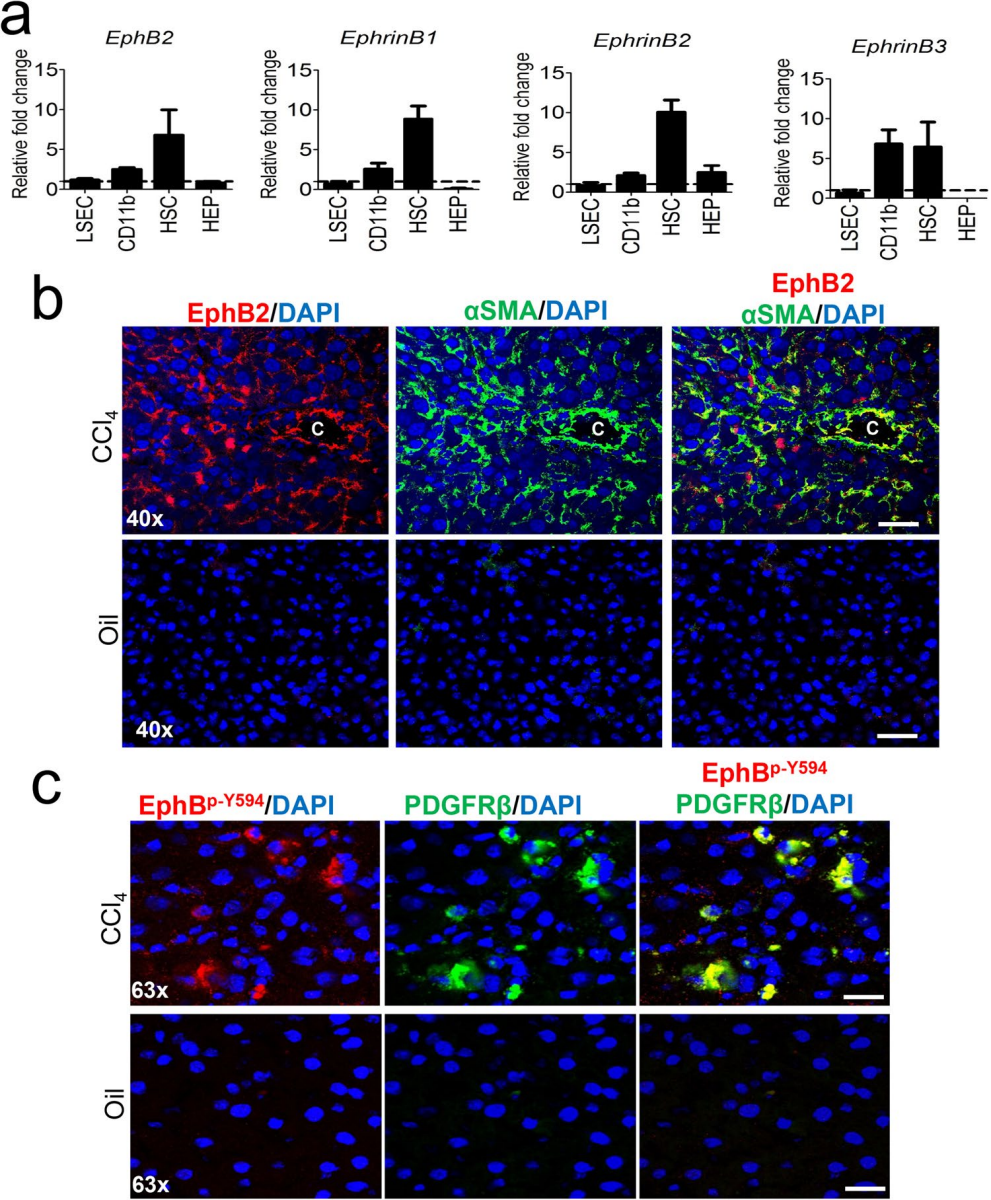
Expression of EphB2 increases and is activated on HSCs after chronic CCl_4_ -induced liver injury. (**a**) Isolated cell fractions from livers of mice subjected to chronic CCl_4_ injections were analyzed for *EphB2*, *Ephrin-B1*, *Ephrin-B2* and *Ephrin-B3* mRNA levels using RT-qPCR. Results are shown as fold change compared to liver cell fractions obtained from vehicle-treated controls. Error bars represent mean ± SEM.; n = 6 animals; CD11b = macrophages, LSEC = Liver sinusoidal endothelial cells, HEP = Hepatocytes and HSCs = Hepatic stellate cells. (**b**) OCT liver sections from C57BL/6 J mice chronically injected with CCl_4_ or vehicle (oil) controls were stained with EphB2 (red), αSMA (green) and DAPI/DNA (blue) and analyzed using confocal microscopy. Scale bar = 100 µm, “C” denotes the central vein. All images are representative of 5 mice per group. (**c**) OCT liver sections from C57BL/6 J mice chronically injected with CCl_4_ or vehicle controls were stained with phospho-EphB1/EphB2-Y594 (red), PDGFRβ (green) and DAPI/DNA (blue) and analyzed using confocal microscopy. Scale bar = 50 µm. All images are representative of 5 mice per group.

To determine which cell type within the liver contributes to the observed increase in EphB2 during liver fibrosis we carried out cell fractionation on livers from CCl_4_-treated and oil-treated mice. We determined mRNA levels of *EphB2* and *Ephrin-B* ligands in each cell type isolated (liver sinusoidal endothelial cells (LSEC), hepatocytes (HEP), HSCs and macrophages). No major change in *EphB2* mRNA levels was observed in LSEC or HEP during CCl_4_-induced liver fibrosis (Fig. 2a). However, relative to livers from oil-treated control mice, *EphB2* mRNA was upregulated in HSCs ~7-fold and CD11b macrophages ~2 fold isolated from CCl_4_-treated mice. Interestingly, whilst *EphrinB3* mRNA was upregulated in both HSCs and CD11b+ macrophages from CCl_4_-treated mice relative to oil treated control mice (~6 fold in both cell types), expression of *EphrinB1* and *EphrinB2* mRNA followed the same trend as *EphB2* with respect to being highly upregulated in HSCs (~10-fold for both genes) compared to CD11b+ macrophages (Fig. 2a). Collectively this data suggest that HSCs are potentially the primary cell type that upregulate transcription of *EphB2* and *Ephrin-B ligands* in CCl_4_-induced liver fibrosis. Confirmation of the *EphB2* transcriptome profiles was provided by immunofluorescence analysis of liver sections from oil and CCl_4_-treated mice stained with EphB2 and αSMA antibodies. Most of EphB2 staining co-localized with α-SMA (Fig. 2b) suggesting that activated HSCs do upregulate EphB2 during liver fibrogenesis in this model. This finding is significant because HSCs are the main cellular source of collagen in CCl_4_-induced hepatic fibrogenesis^4,28,29^.

To determine whether EphB2/Ephrin-B system is activated during liver fibrogenesis, we stained liver sections from CCl_4_-treated mice with an antibody that detect phosphorylated EphB1/EphB2 as a read-out for activated EphB2. These sections were co-stained with platelet derived growth factor receptor-β (PDGFRβ), another fibrogenic marker of activated HSCs^30^.

We noted an increase in phospho-EphB1/EphB2 that strongly co-localized with PDGFRβ expression in CCl_4_-treated mice (Fig. 2c). This data suggest that EphB2 receptor signaling (forward signaling) is induced on activated HSCs in the fibrotic liver microenvironment.

### Expression of EphB2 on HSCs is associated with the differentiation of quiescent HSCs into fibrogenic myofibroblasts *in vitro*

**Figure 3:**
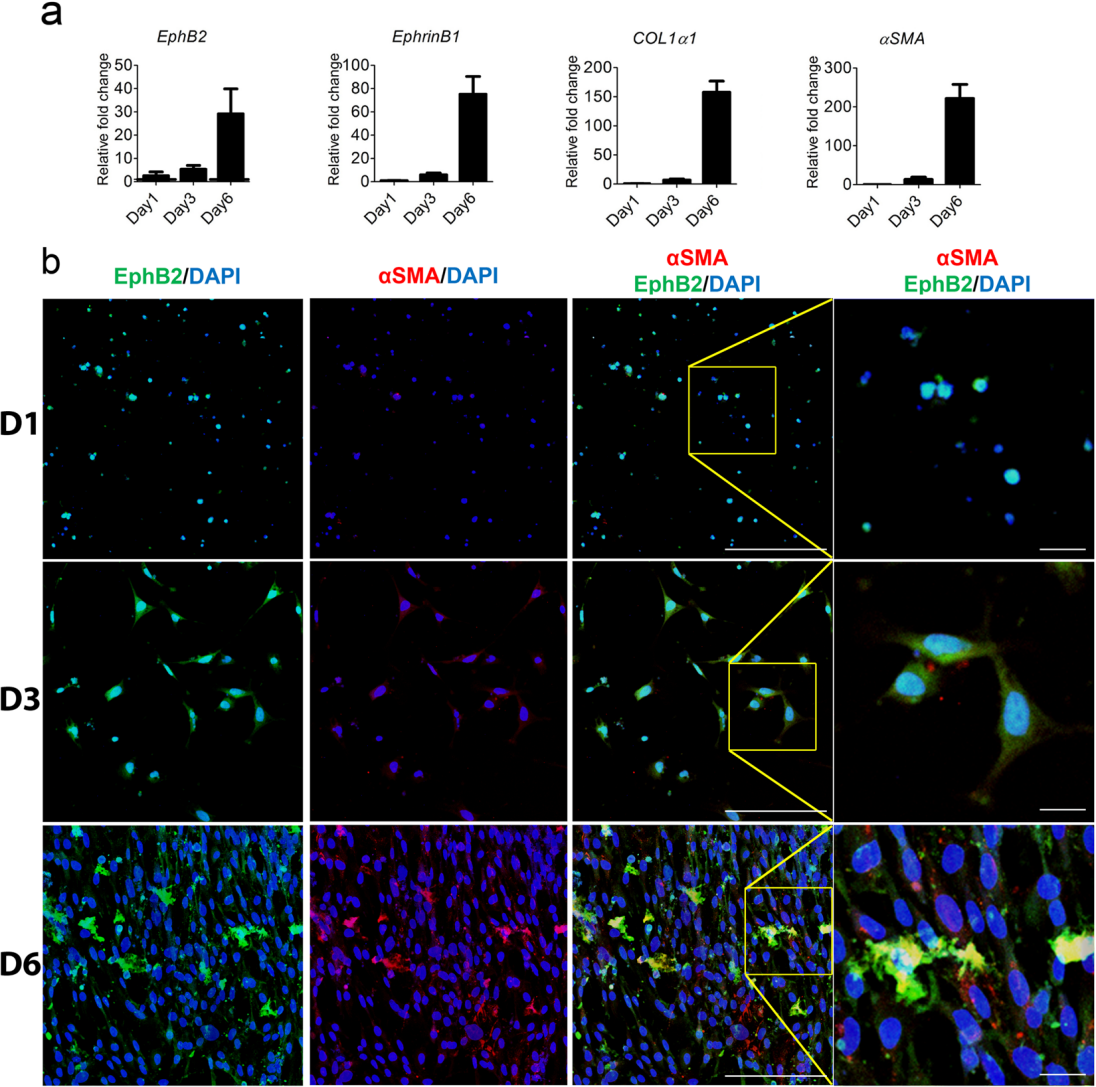
Expression of EphB2 is correlated with αSMA production on culture-activated HSCs. (**a**) Primary HSCs isolated from the livers of C57BL/6 mice were culture-activated on uncoated plastic dishes for 6 days. *EphB2*, *EphrinB1*, *COL1α1* and *α-SMA* mRNA levels were measured by RT-qPCR at different time points. Results are shown as fold change compared to day 0 HSCs for each target transcript. Error bars represent mean ± SEM from 4 independent experiments. (**b**) Primary HSCs isolated from the livers of C57BL/6 mice were culture-activated on uncoated plastic dishes for 6 days. Cells were fixed at various time points and stained for EphB2 (green), αSMA a marker of activated HSCs (red) and DAPI/DNA (blue). EphB2/αSMA co-localization signal appears orange in the images. Far-right panels show 3× magnified area from yellow squares in the overlay (EphB2/αSMA) images). Representative images on various days are shown. D1 = day 1, D3 = day 3 and D6 = day 6. Magnification 200×, Scale bar = 200 µm.

It is well established that when cultured into plastic dishes, quiescent HSCs transdifferentiate into fibrogenic myofibroblasts as evidenced by their change in morphology and increased expression of fibrogenic molecules including COL1α1 and α-SMA^31,32^. We next investigated the possibility of a temporal correlation between expression of EphB2 and one of its ligands Ephrin-B1 with differentiation of HSCs *in vitro*. We noted a gradual increase in *EphB2* and *αSMA* mRNA and protein levels in HSCs after three and six days in culture (Fig. 3a,b). Interestingly, *Ephrin-B1* and *COL1α1* mRNA were also elevated in these same culture-activated HSCs. This data shows that expression level of EphB2 is correlated with the differentiation of quiescent HSCs into fibrogenic myofibroblasts *in vitro*.

### EphB2 deficiency attenuates CCl_4_-induced hepatic fibrosis in mice

**Figure 4:**
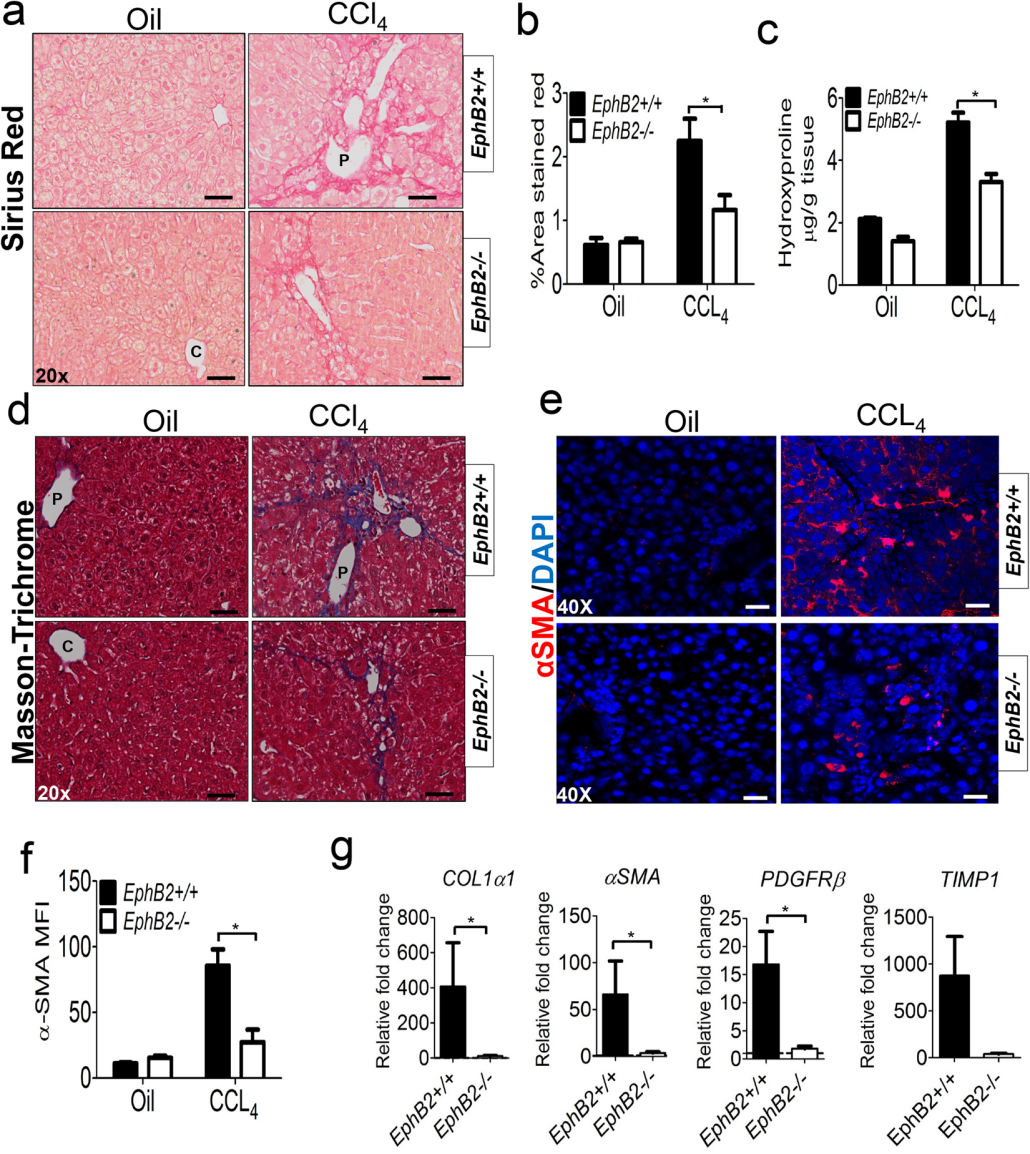
EphB2-deficient mice are protected from CCl_4_-induced hepatic fibrosis. *EphB2−/*− mice and wild type littermate control mice (WT) were chronically injected with CCl_4_ twice a week for 6 weeks and sacrificed 72 h after receiving the last dose of CCl_4_. (**a**) Representative microscopic images showing picrosirius red staining of the liver sections used to detect collagen deposition. Scale bar = 200 µm; “C” denotes the central vein and “P” denotes the portal vein. (**b**) ImageJ quantification of picrosirius red in the livers of CCl_4_ injected *EphB2−/*− and WT littermate mice. Data are mean ± SEM. n = 5 animals per group, *p < 0.05. (**c**) Hydroxyproline quantification of collagen deposition in the liver of *EphB2−/*− and WT littermate mice subjected to chronic injections of CCl_4_. Data are mean ± SEM. n = 5 animals per group, *p < 0.05. (**d**) Representative microscopic images showing Masson-trichrome staining for evaluation of collagen deposition in the liver sections of *EphB2−/*− and WT littermate mice subjected to chronic injections of CCl_4_. Scale bar = 200 µm; “C” denotes the central vein and “P” denotes the portal vein. (**e**) Representative microscopic images showing immunofluorescence staining of α-SMA a marker of HSC activation in the liver sections of *EphB2−/*− and WT littermate mice subjected to chronic injections of CCl_4_. Scale bar = 100 µm. All images are representative of 5 mice per group. (**f**) ImageJ quantification of α-SMA immunofluorescence taken across ten randomly selected areas of liver sections obtained from CCl_4_ injected *EphB2−/*− and WT littermates mice. MFI = Mean Fluorescence Intensity. Data are mean ± SEM. n = 5 animals per group, *p < 0.05. (**g**) Quantitative RT-PCR for gene expression analysis of the profibrotic markers *COL1α1*, *α-SMA*, *PDGFRβ* and *TIMP-1* from total RNA prepared from the liver of *EphB2−/*− and WT littermate mice subjected to chronic injections of CCl_4_. Data are mean ± SEM. n = 5 animals per group, *p < 0.05.

To determine whether EphB2 expression is required for the development of liver fibrosis, we made use of EphB2-deficient^19^ and littermate control mice that were chronically treated with CCl_4_ twice a week for 6 weeks. Compared to similarly treated wild type littermate controls, CCl_4_-treated *EphB2−/*− mice had reduced liver fibrosis (Fig. 4). Sirius red and Masson-trichrome staining of liver sections demonstrated a reduction of collagen deposition (*P* < 0.05) in the livers of CCl_4_-treated *EphB2−/*− mice compared to CCl_4_- treated littermate controls (Fig. 4a,b,d). This observation was further supported by hydroxyproline quantitation of collagen which was reduced in CCl_4_-treated *EphB2−/*− mice compared to CCl_4_-treated littermate controls (Fig. 4c). We also observed a drastic reduction in mononuclear cells infiltrating in the liver of CCl_4_-treated *EphB2−/*− mice compared to CCl_4_- treated littermate controls (Supplementary Fig. S3).

Alpha-smooth muscle actin (α-SMA) has been suggested to be a potential marker of liver activated HSCs since its expression increases dramatically during differentiation of quiescent HSCs into myofibroblasts. Following α-SMA immunofluorescence staining of liver sections, we noted a significant reduction (*P* < 0.05) of α-SMA expression in the livers of CCl_4_-treated *EphB2−/*− mice compared to CCl_4_- treated littermate controls (Fig. 4e,f). This observation was further supported by transcriptional analyses showing a significant reduction (*P* < 0.05) of the mRNA levels of *αSMA* as well as some major pro-fibrotic markers genes namely *COL1α1*, *PDGFRβ* and *tissue inhibitor of metalloproteinase* (*TIMP1)* in the livers of CCl_4_-treated *EphB2−/*− mice compared to CCl_4_- treated littermate controls (Fig. 4g). Collectively, this data demonstrates involvement of EphB2 receptor in the pathological mechanism of hepatic fibrosis in this model.

### EphB2 is required for a robust hepatic inflammatory response during liver injury

**Figure 5:**
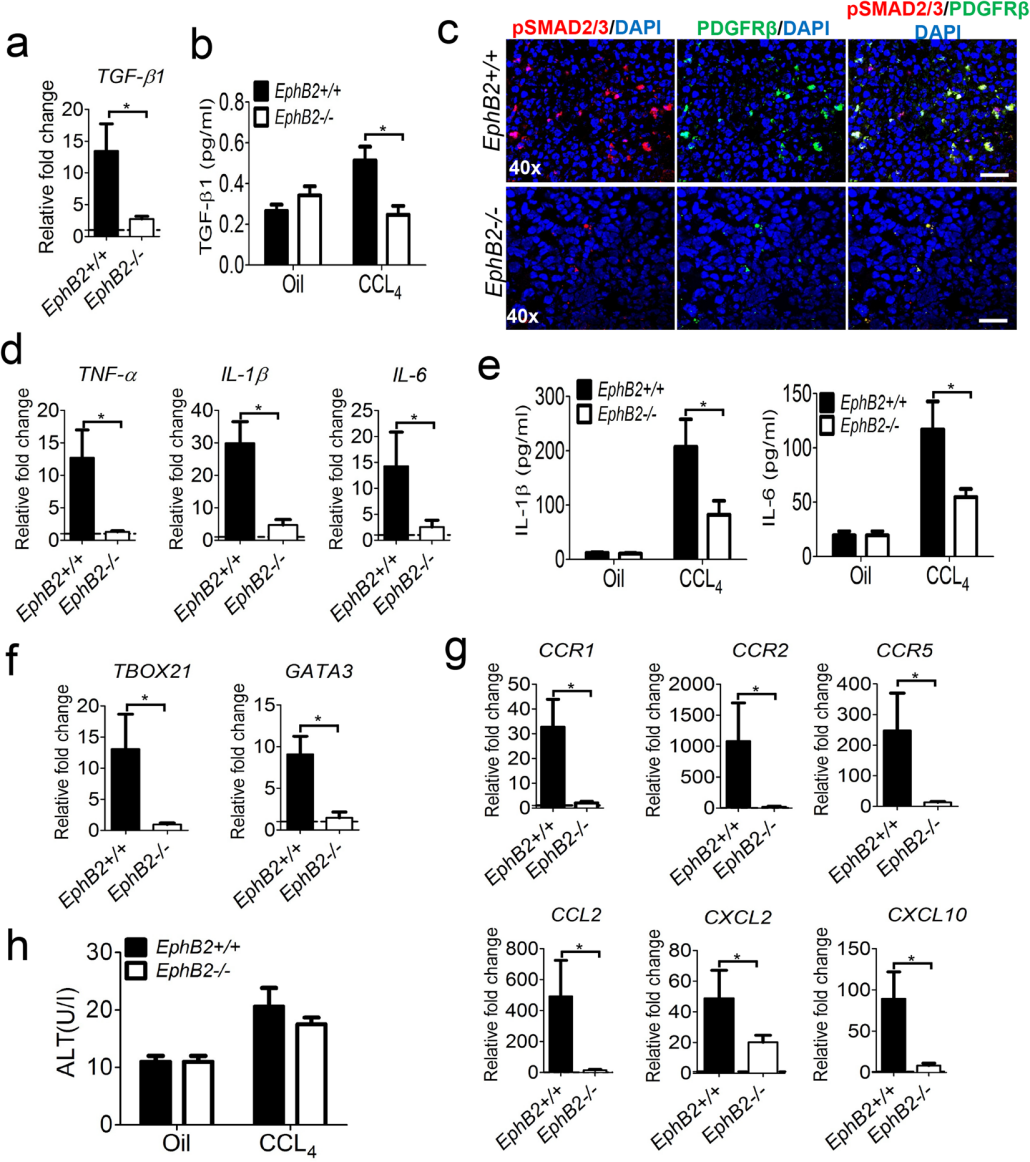
EphB2-deficiency reduces TGF-β1 signaling and inflammation in the liver during CCl_4_-mediated hepatic injury. *EphB2−/*− mice and wild type littermates (WT) were chronically injected with CCl_4_ twice a week for 6 weeks and sacrificed 72 h after receiving the last dose of CCl_4_. (**a**) *TGF-β1* mRNA levels were analyzed by RT-qPCR in livers of *EphB2−/*− and WT littermate mice subjected to chronic injections of CCl_4_. Results are shown as fold change compared to vehicle-treated controls. Error bars represent mean ± SEM. n = 5 animals, *p < *0.05*. (**b**) Total plasma TGF-β1 level determined using a Singleplex bead Luminex® assay in *EphB2−/*− and WT littermate mice subjected to chronic injections of CCl_4_. Data are mean ± SEM. n = 5 animals, *p < *0.05*. (**c**) Representative immunofluorescence images of liver tissue sections stained for phospho-SMAD2/SMAD3 (red) a marker of canonical TGF-β1 pathway activation and PDGFRβ (green) a marker for HSCs activation in the liver of *EphB2−/*− and WT littermate mice subjected to chronic injections of CCl_4_. Scale bar = 100 µm. All images are representative of 5 mice per group. (**d**) *TNF-α*, *IL1-β* and *IL-6* mRNA levels were analyzed by RT-qPCR in livers of *EphB2−/*− and WT littermate mice subjected to chronic injections of CCl_4_. Results are shown as fold change compared to vehicle-treated controls. Error bars represent mean ± SEM. n = 5 animals. *p < *0.05* (**e**) Total plasma for IL1-β1 and IL-6 levels determined using a Singleplex bead Luminex® assay in *EphB2−/*− and WT littermate mice subjected to chronic injections of CCl_4_. Data are mean ± SEM. n = 5 animals, *p < *0.05*. (**f**) *Tbox-21* and *GATA-3* mRNA levels were analyzed by RT-qPCR in livers of *EphB2−/*− and WT littermate mice subjected to chronic injections of CCl_4_. Results are shown as fold change compared to vehicle-treated controls. Error bars represent mean ± SEM. n = 5 animals, *p < *0.05*. (**g**) Gene expression analysis of the profibrogenic chemokines receptors *CCR1*, *CCR2* and *CCR5* and the chemokine ligands *CCL2*, *CXCL2* and *CXCL10* mRNA levels assessed by RT-qPCR in the liver of EphB2*−/*− and WT littermate mice subjected to chronic injections of CCl_4_. Data are mean ± SEM. n = 5 animals, *p < *0.05*. (**h**) Plasma ALT level in *EphB2−/*− and WT littermate mice subjected to chronic injections of CCl_4_. Data are mean ± SEM. n = 5 animals.

Fibrosis is usually accompanied by the release of pro-fibrotic and inflammatory cytokines/chemokines in the affected organs, with transforming growth factor-β1 (TGF-β1) being a principal mediator of fibrogenesis^33^. Given that CCl_4_-treated *EphB2−/*− mice developed less fibrosis compared to CCl_4_-treated littermates, we hypothesized that this observation may be related to a defective inflammatory response in the liver following injury. We noted that *TGF-β1* mRNA was downregulated in the livers of CCl_4_-treated *EphB2−/*− mice (Fig. 5a) and this was correlated by a decrease in peripheral total TGF-β1 in the plasma (Fig. 5b).

TGF-β1 binds to type I and type II TGFβR serine/threonine kinases on the surface of HSCs which then triggers the phosphorylation of SMAD2 and SMAD3 in the cytoplasm that will then form a complex with SMAD4. This SMAD complex translocate in the nucleus and recognizes SMAD-binding elements on the genome; regulating expression of pro-fibrotic genes^33^. Given that transcription of *TGF- β1* was reduced in the livers of CCl_4_-treated *EphB2−/*− mice compared to littermates, we determined whether the reduction in liver fibrosis in CCl_4_-treated *EphB2−/*− mice could also be resulting from a compromised phosphorylation of SMAD complex on HSCs. Immunofluorescence imaging of liver sections stained with antibodies for phosphoSMAD2/SMAD3 and PDGFR-β show a markedly reduced phosphoSMAD2/SMAD3 and PDGFR-β protein levels in the livers of CCl_4_-treated *EphB2−/*− mice compared to CCl_4_- treated littermate controls (Fig. 5c). This data is consistent with the reduction of liver fibrosis we observed in *EphB2−/*− mice following chronic CCl_4_ liver injury.

Next, we determined whether the reduction of liver fibrosis in CCl_4_-treated *EphB2−/*− mice was also associated with a decrease in pro-inflammatory cytokines and chemokines. RNA transcripts for tumor necrosis factor-α (*TNF-α)*, interleukin(*IL)-1β* and *IL-6* genes were all significantly downregulated in the livers of CCl_4_-treated *EphB2−/*− mice compared to CCl_4_-treated littermate control animals (Fig. 5d) and this was mirrored by a significant reduction in the plasma level of total IL-1β and IL-6 (Fig. 5e). The levels of *T-box 21* and *GATA-3* mRNA, representative of Th1 and Th2 transcriptions factors respectively, were also significantly decreased in fibrotic livers of *EphB2−/*− mice compared to intact littermate controls (Fig. 5f). Thus, it is possible that EphB2 might be playing a role in the maintenance of both Th1-related inflammation and Th2-related fibrogenesis.

Several chemokine/chemokine receptor pathways have been linked to the pathogenesis of liver fibrosis by promoting the recruitment of monocytes/macrophages to the site of liver injury. Consistent with our data showing that EphB2 deficiency impacts the recruitment of immune cells to the site of liver injury (Supplementary Fig. S3), transcripts for the chemokine receptors (*CCR1*, *CCR2* and *CCR5*) and chemokine ligands (*CCL2*, *CXCL2* and *CXCL10*) were all downregulated in the livers of CCL_4_-treated *EphB2−/*− mice compared to CCl_4_-treated littermates (Fig. 5g). Thus, EphB2 might be required for the substantial hepatic inflammatory response that accompanies fibrosis.

Analysis of liver enzyme damage indicated a trend toward a reduction of plasma ALT in CCl_4_-treated *EphB2−/*− mice compared to CCl_4_-treated *EphB2*+/+ mice although this trend did not reach statistical significance (Fig. 5h).

### EphB2 deficiency mitigates *in vitro* differentiation of HSCs into myofibroblasts

**Figure 6:**
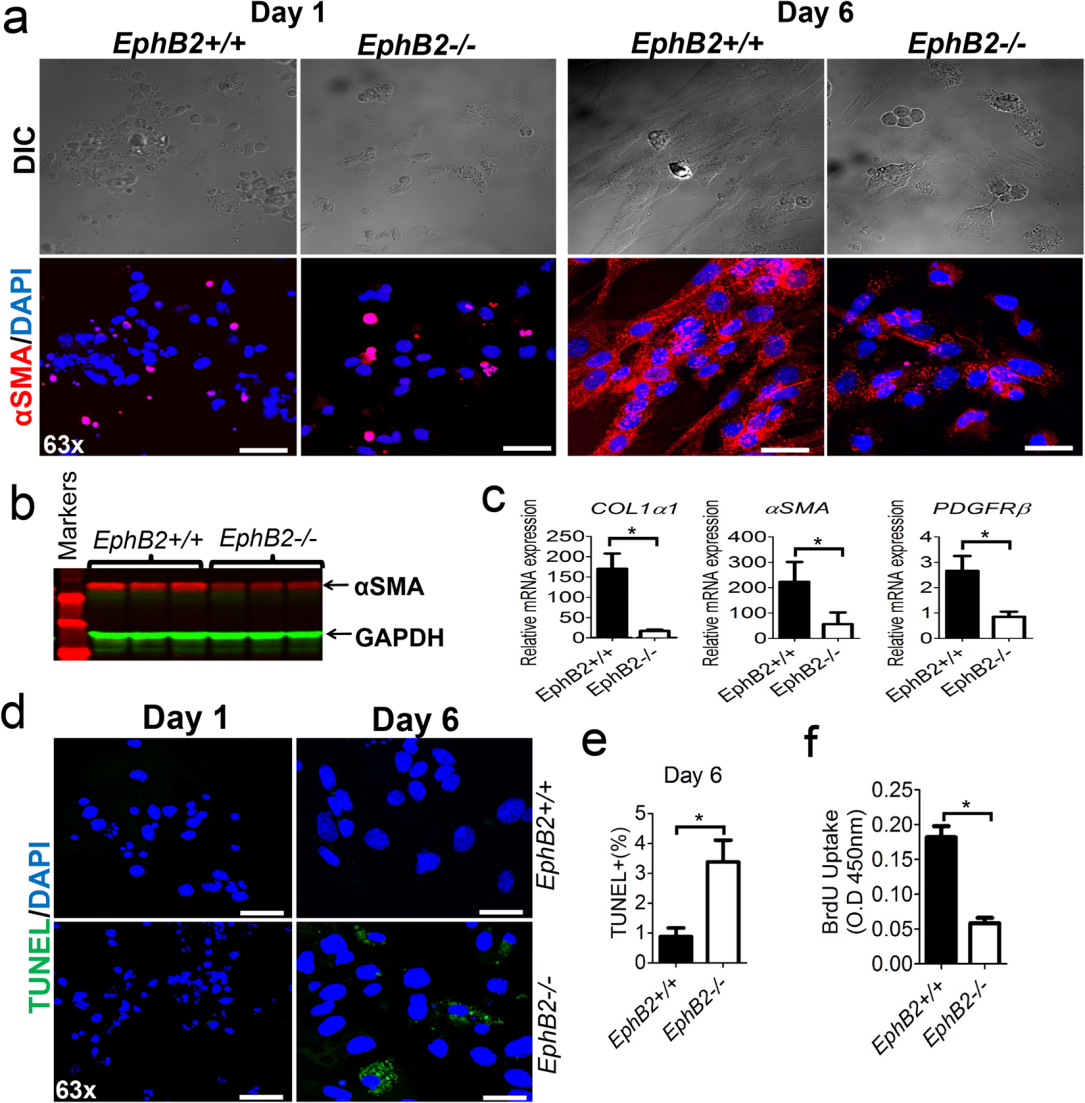
HSCs isolated from EphB2-deficient mice have reduced activation compared to those isolated from intact littermate controls. Purified HSCs from *EphB2*+/+ and *EphB2−/*− mice were cultured for 6 days in DMEM medium supplemented with 20% serum. Myofibroblasts activation was assessed by immunofluorescence, western blot and RT-qPCR. (**a**) Representative immunofluorescence images showing expression of α-SMA (red) and DAPI/DNA (blue) from *EphB2*+/+ and *EphB2−/*− myofibroblasts obtained after one and six days of culture. Scale bar = 50 µm. (**b**) Western blot of αSMA from *EphB2*+/+ and *EphB2−/*− myofibroblasts obtained after 6 days of culture. (**c**) Gene expression analysis of the profibrogenic markers *COL1α1*, *α-SMA* and *PDGFRβ* assessed by RT-qPCR in day 6 myofibroblasts from *EphB2−/*− and WT littermate control mice relative to day 0 purified HSCs. Error bars represent mean ± SEM from 4 independents experiments. *p < *0.05*. (**d**) Representative images of TUNEL assay for detection of apoptosis in day 1 and day 6 cultured myofibroblasts from *EphB2−/*− and WT littermate control mice. Scale bar = 50 µm. (**e**) Quantitation of apoptosis after 6 days of cultured of myofibroblasts from *EphB2−/*− and WT littermate control mice. Error bars represent mean ± SEM from 4 independents experiments. *p < *0.05*. (**f**) BrdU proliferation assay for culture-activated HSCs isolated from *EphB2−/*− and WT littermate control mice. These data are representative of at least four independents experiments in each case. Error bar represent Mean ± SEM. *p < *0.05*.

Having shown that EphB2 expression is elevated on HSCs during liver fibrosis and that it is required for TGF-β1 signaling and CCl_4_-induced hepatic fibrosis in mice, we next hypothesized that EphB2 deficiency could dampen the differentiation of HSCs into fibrogenic myofibroblasts. To test this hypothesis, we isolated HSCs from *EphB2*+/+ and *EphB2−/*− and allowed them to differentiate into myofibroblasts *in vitro*. After six days of culture, we noted a sub-optimal differentiation of quiescent HSCs into myofibroblasts from *EphB2−/*− mice compared to HSCs isolated from littermate controls. Immunofluorescence staining of day 6 culture-activated HSCs with anti-α-SMA antibody demonstrated that *EphB2−/*− HSCs have less stress fibers and also incorporated less α-SMA protein into stress fibers compared to *EphB2*+/+ HSCs (Fig. 6a). Immunoblotting of day 6 culture-activated HSCs with anti-α-SMA antibody further supported a defective activation of HSCs isolated from *EphB2−/*− mice compared to HSCs isolated from *EphB2*+/+ mice (Fig. 6b). In addition, we noted a reduction in mRNA levels of *α-SMA*, *COL1-α1* and *PDGFR-β* in *EphB2−/*− culture-activated HSCs compared to HSCs isolated from littermate controls (Fig. 6c). Altogether, our data imply that culture-activated HSCs poorly transdifferentiate into fibrogenic myofibroblasts in the absence of EphB2.

To ascertain whether this sub-optimal activation of *EphB2−/*− myofibroblasts could be due to their susceptibility to apoptosis *in vitro* we used a TUNEL assay. We observed an increased in apoptosis in day 6 culture-activated HSCs isolated from *EphB2−/*− mice compared to HSCs isolated from *EphB2*+/+ mice (Fig. 6d,e). We also noted that proliferation of fibrogenic myofibroblasts, as assessed by BrdU incorporation, was indeed attenuated in the absence of EphB2 (Fig. 6f). Taken together our data suggest that in the absence of EphB2, culture-activated HSCs are more prone to apoptosis and have a defective proliferation rate *in vitro*.

### Elevated apoptosis in the liver of *EphB2−/*− mice following CCl_4_ injury

**Figure 7:**
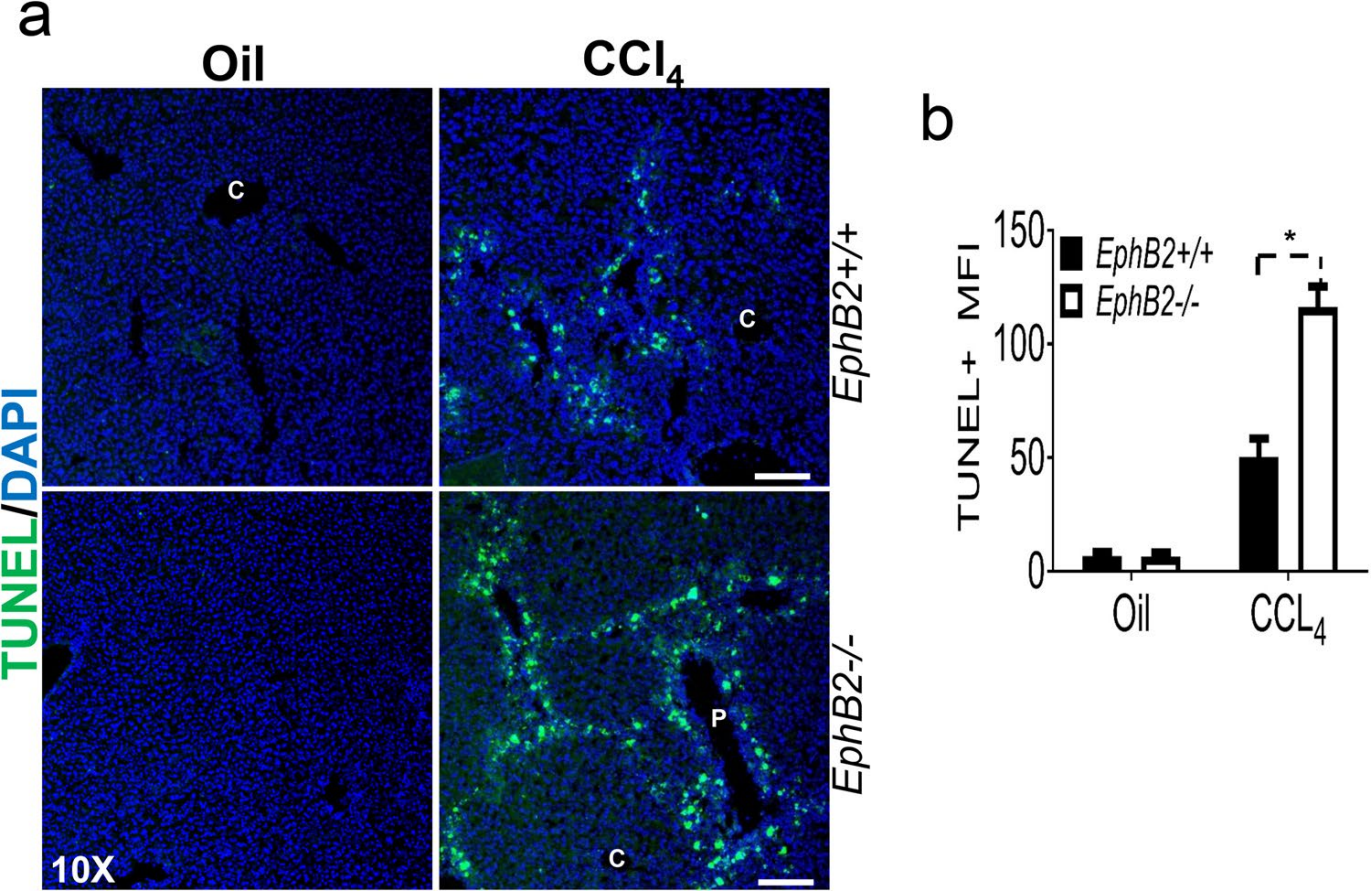
Increased apoptosis in the livers of *EphB2−/*− mice subjected to CCl_4_-induced fibrogenic injury. *EphB2−/*− mice and wild type littermates (WT) were chronically injected with CCl_4_ twice a week for 6 weeks and sacrificed 72 h after receiving the last dose of CCl_4_. (**a**) Representative microscopic images showing immunofluorescence staining for TUNEL in the liver sections of *EphB2−/*− and WT littermate mice subjected to chronic injections of CCl_4_. Scale bar = 400 µm. All images are representative of 3 mice per group. (**b**) ImageJ quantification of TUNEL immunofluorescence taken across ten randomly selected areas of liver sections obtained from CCl_4_ injected *EphB2−/*− and WT littermate mice. Data are mean ± SEM. n = 3 animals per group. MFI = Mean Fluorescence Intensity, *p < *0.05*.

To counteract fibrosis-inducing insults in the liver, apoptosis is a key mechanism to thwart fibrogenesis. Since CCl_4_-treated *EphB2−/*− mice have reduced fibrosis relative to wild type littermate control animals, we hypothesized that this could be the result of an increased in apoptosis of fibrogenic cells in the liver. TUNEL staining of liver sections of CCl_4_-treated *EphB2−/*− mice displayed a significant elevation in apoptosis compared to CCl_4_- treated littermate controls (Fig. 7a,b) supporting a role for EphB2 in suppressing apoptosis and repair mechanisms upon chronic liver injury. This may explain the limited fibrosis that occurs in *EphB2−/*− mice upon CCl_4_-induced chronic liver injury.

## Discussion

Fibrosis is a complex pathology affecting various organs including the liver, lung, heart, kidney, bone, skin, eye and even the central nervous system (CNS)^34^. In the present study, we have shown that EphB2 is potentially a critical regulator of liver inflammation and fibrogenesis. In human, EphB2 was initially found to be highly upregulated in hepatocellular carcinoma, the end-stage of liver fibrosis/cirrhosis^18^. Our current work in mouse models of hepatic fibrosis have identified *EphB2* mRNA transcripts as the most upregulated of the five *EphB* receptors in fibrotic livers of CCl_4_ and *MDR2*-null mice. This appears to be a general trend in mice as transcription of EphB2 was also elevated in STAM™ mice, a newly developed animal model of non-alcoholic steatohepatitis (NASH) compared to control animals (data not shown). Furthermore, our human biopsy data showing that EphB2 expression is upregulated in liver cirrhosis (Supplementary Fig. S2) indicates that these observations have relevance to human liver fibrosis. All this evidence, coupled to our previous work showing increase EphB2 expression in the liver of malaria-infected mice^26^, collectively suggest that EphB2 upregulation is likely a basic response to chronic liver injury-inducing fibrosis, regardless of the initiating agent. As such, understanding the mechanisms by which EphB2 promote fibrosis would potentially enable development of novel EphB2-based anti-fibrotic therapy.

Hepatic fibrosis is the result of a chronic inflammatory process affecting various liver cell subtypes^6^. The inflammatory insult generates a microenvironment that promotes the synthesis and deposition of extracellular matrix (ECM) proteins by activated HSCs, the primary source of ECM proteins in the liver. It is also well documented that in order to efficiently produce ECM, quiescent HSCs must undergo a transformation into fibrogenic myofibroblasts^34^. Eph/Ephrin signaling is known to regulate cell proliferation, migration and tissue remodelling^35^ which are core events occurring during the reprogramming of HSCs into fibrogenic myofibroblasts both *in vitro* and *in vivo*^4^. Our data support a role specifically for EphB2 in these processes. Although EphB2-deficient mice appear “normal”, and are fertile, they do have an inherent defect especially in establishing a precise organization of tissues during the development of embryonic organs^19,36^. Furthermore, an improper migration or seeding of immune cells within lymphoid organs like the thymus has been observed in these mice^37^. Interestingly, developmental pathways (like the Eph/Ephrin family) involved in tissue patterning during embryogenesis appear to be modulated in organs fibrosis^24^. Consequently, we were able to show that the developmental guidance cue EphB2 is actively signaling in the fibrotic liver. Furthermore we have shown that modulation of EphB2 expression predominantly occurs in HSCs (Fig. 2) and transdifferentiation of quiescent HSCs into fibrogenic myofibroblasts requires EphB2 (Fig. 6). It is likely that EphB2 expression in other cells within the liver microenvironment like immune cells (macrophages etc), LSECs and HEPs also regulate hepatic fibrogenesis. Future studies will tackle this point by targeted-deletion of EphB2 in various liver cell type during fibrogenesis.

Reprogramming of HSCs requires TGF-β1 signaling. EphB2 has previously been identified as a new target for TGF-β signaling, influencing migration and invasion of breast cancer cells^38^ and in a separate study the positioning of proliferating cells within the colonic crypts of intestine partly depends on EphB2/SMAD3 signaling^39^. Here we show that in the absence of EphB2 hepatic *TGF-β1* mRNA and plasma levels were downregulated in *EphB2−/*− mice following CCl_4_ exposure when compared to intact littermate control mice (Fig. 5). Consistent with these observations we also noted a reduction in phosphorylated SMAD2/SMAD3 signaling molecules downstream TGFβRI/II activation pathway in the livers of *EphB2−/*− mice following CCl_4_ exposure. Thus, it is plausible that EphB2 promotes the differentiation of HSCs into fibrogenic myofibroblasts by modifying TGB- β1 production and/or signaling.

Other than links to TGF-β1 signaling relatively little is known about the molecular mechanisms or machinery used by EphB2 to facilitate the formation of myofibroblasts from HSCs precursors. However it is tempting to speculate that the cytoplasmic domain of EphB2 containing a tyrosine kinase domain and a PDZ-binding motif can serve as docking site for mechanosensitive factors such as Yes-associated protein 1 (YAP1) and the transcriptional coactivator with PDZ-binding motif (TAZ) which are core mediators of myofibroblasts activation during fibrogenesis^40,41^. Indeed the same intracellular domain of EphB2 closely interacts with myosin1b, a motor protein implicated in the redistribution of myosin II in actomyosin fibers that facilitate changes in cell morphology, a biological process observed during myofibroblasts biogenesis^42^.

Since liver inflammation and fibrosis are interconnected^6^, the reduction in the degree of fibrosis in EphB2-deficient animals chronically exposed to CCl_4_ led us to question whether these mice also have a defective inflammatory response, in addition to the reduction in TGF-β1. Indeed we noticed that EphB2-deficient mice have a drastic reduction of proinflammatory cytokines and chemokines responsible for the maintenance of a profibrotic state in injured liver^43,44^. Our data corroborate recent findings linking EphB2 activation with the regulation of TNFα/NF-κB axis in hepatocytes^26^ as well as in CNS diseases^45^ and this strengthens the possible crosstalk between both the EphB2/NF-κB (pro-inflammatory state) and EphB2/TGF-β (pro-fibrotic state) signaling axes. Further research is needed to understand how activation of the EphB2/EphrinB signaling modulate both the NF-κB and TGF-β signaling pathways.

EphB2 receptor could potentially be targeted to induce regression of liver fibrosis, a process that is characterized by the reduction in the number of activated HSCs/myofibroblasts due to a combination of cellular senescence as well as apoptosis in HSCs^46^. Indeed, we noted a consistent reduction of HSCs expressing α-SMA and PDGFR-β (Figs 4e and 5c) as well as a drastic increase in apoptosis in EphB2-deficient CCl_4_-exposed mice compared to littermate controls (Fig. 7). This data supports a role for EphB2 deficiency in limiting fibrosis and potentially promoting repair of liver tissue in the face of chronic fibrotic injury.

In summary, this study highlights EphB2 as a key fibrogenic molecule involved in the development and progression of hepatic fibrosis. Pending further validation with specific pharmacological inhibitor, EphB2 could potentially be a novel therapeutic target for the treatment of liver fibrosis/cirrhosis.

## Methods

### Mice

Female *MDR2-null* (*abcb4−/*−)^27^, FVB control and C57BL/6 wild type (WT) mice aged 6–8 weeks were bred in-house or purchased from The Jackson laboratory (Bar Harbor, ME, USA). Female *EphB2−/*− mice^19^ on a C57BL/6 background were re-derived (a kind gift from Dr. Jonathan Gibbins, University of Reading, UK) and bred in-house under a heterozygous breeding system. Mice were given water and food (LabDiet, MO, USA: chow 5001) *ad libitum* and housed under standard conditions. All experiments were approved and carried out according to protocols approved by the Institutional Animal Care and Use Committee of the University of Utah and Emory University following the National Institute of Health guidelines for the care and use of laboratory animals.

### CCl_4_ model of hepatic fibrosis

Mice aged 6–8 weeks were injected intraperitoneally with 2 µl/g of CCl_4_ (Sigma) (adjusted to 10% concentration in olive oil) or olive oil twice a week for 6 weeks. Mice were sacrificed by CO_2_ inhalation 72 hours after the last dose of CCl_4_ and the livers removed and processed for further analysis. For an acute CCl_4_-mediated liver injury, mice were administered one dose (24-hour), three doses (three times a week) and six doses (three times a week for two weeks) of 10% CCl_4_ or vehicle (oil) and sacrificed by CO_2_ inhalation 24 h later.

### *MDR2*-null model of biliary fibrosis

The *MDR2*-null model of biliary fibrosis has been described elsewhere^27^. Briefly, *MDR2−/*− mice and controls on FVB background were fed a normal rodent laboratory diet for 25 weeks until which they were sacrificed and the livers excised and processed for further analysis.

### Hepatic stellate cell isolation

Livers from individual mice were sequentially perfused through the inferior vena cava with Krebs-Ringer bicarbonate buffer followed by 0.3% pronase (Roche) and 0.3 mg/ml type IV collagenase (Sigma). Then excised, minced with scissors and further digested with 0.1 mg/ml collagenase/dispase (Roche) supplemented with 0.008% deoxyribonuclease I (Roche). The cells suspensions were shaken (250 rpm) at 37 °C for 20 min and filtered through a 70 µm sterile cell strainer. To remove hepatocytes, the cells suspensions were centrifuged at 90 g for 5 min, and the supernatant collected. The latter was further centrifuged at 400 g for 10 min at 4 °C to collect the nonparenchymal fraction. Collected cells were resuspended in 5 ml 17% OptiPrep™ gradient density (Sigma) and 5 ml of 11.5% OptiPrep™ was gently added followed by 2 ml of Gey’s balanced salt solution GBSS (Sigma). The cell suspension was centrifuged at 1400 g for 20 min at 4 °C and the cells layered between the 11.5% OptiPrep™ and GBSS representing HSCs were carefully aspirated, resuspended in completed medium (DMEM supplemented with 5mM L-Glutamine, 20 mM HEPES, 1 nM sodium pyruvate, 1× β-mercaptoethanol, 1× penicillin/streptomycin and 20% FCS). The cells were centrifuged at 400 g for 10 min, counted and plated on either a 96-well, 24-well plate (Nunc) or 8-well chamber slides for microscopy. These primary HSCs were cultured for at least 6 days at 37 °C in a CO_2_ incubator to progressively differentiate into myofibroblasts.

### Isolation of other nonparenchymal cell fraction

This was partly achieved following the procedure outline above for isolation of HSCs and completed as described elsewhere^28^. Briefly after the livers were perfused and the hepatocytes isolated as described above, the nonparenchymal cells fraction (consisting of HSCs, portal fibroblasts, macrophages, bone marrow cells, endothelial cells, etc) was recovered. HSCs were isolated as described above. Macrophages and endothelial cells were isolated by gradient centrifugation in 15% Nycodenz followed by magnetic sorting with anti-CD11b and anti-CD31 antibodies conjugated beads (Miltenyi Biotec).

### RNA extraction and cDNA synthesis

Cells or tissues were homogenized in RNA Stat60^®^ and total RNA extracted using standard phenol-chloroform protocols followed by DNase treatment of the extracted RNA using RNA-II purification kit (Nachery-Nagel). A total of 100 ng of RNA per sample was converted into cDNA using Superscript II (Life Technologies) at 42 °C for 50 min, 70 °C 15 min, in the presence of 5 uM oligo (dT)_16–18_, 5 mM Dithiothreitol (DTT), 0.5 mM dNTPs (all Life Technologies), 8 U RNAsin (Promega), 50 mM Tris-HCl pH 8.3, 75 mM KCl and 3 mM MgCl_2_. The cDNA was treated with 2.5 U RNase H (Affymetrix) at 37 °C for 20 min to remove any remaining RNA residues.

### Quantitative PCR

Real-time qPCR reactions were performed using Quantitect SYBR Green PCR reagent (Qiagen). PCR amplification was performed with 5 µl cDNA sample (diluted 1:10), 2 µM of each primer, 7 µl of QPCR SYBR green mix and plates run using Applied BioSystems FAST 7000 Sequence detection system (ABI Prism FAST 7000). Primer sequences are shown in Supporting Table S1. Transcripts were normalized to two different housekeeping genes (Ubiquitin and β-actin) and mRNA expression levels calculated using the 2^−ΔΔCt^ method.

### Immunohistochemistry and immunofluorescence

Paraffin-embedded sections were processed for immunohistochemistry following standard protocols^30^. Five micron (5 µm) sections were stained with Hematoxylin and Eosin, picrosirius red, and Masson-trichrome (Sigma) to assess collagen deposition. Quantification of collagen from picrosirius red stained sections was achieved as previously described^26^. For immunofluorescence staining, liver tissues were fixed with 4% paraformaldehyde overnight at 4 °C, immersed in graded sucrose solutions, embedded in OCT (Tissue Tek) and stored at −80 °C until sectioned using a Cryostat (10 µm). HSCs cultured on chamber glass slides were also fixed with 4% paraformaldehyde for 30 min. After blocking, cells and frozen liver sections were incubated overnight at 4 °C with the following primary antibodies: Human/mouse EphB2 clone AF467 (1:50; R&D Systems), phospho-EphB1/EphB2^Y594^ ab61791 (1:200; Abcam), phospho-SMAD2/SMAD3 D27F4 (1:100; Cell signaling); α-SMA clone 1A4 (1:100, Sigma), CD140b (PDGFR-β) APB5 (1:50, eBioscience). Cells or liver sections were then stain with NorthernLights 493^®^ or NorthernLights 577^®^ conjugated secondary antibodies for one hour (1:500 R&D Systems). The nuclei were counterstained with mounting medium containing DAPI (VectorShield). Images were captured using a Carl Zeiss confocal microscope. Digital morphometric measurement of α-SMA were performed using Image J software. Ten random fields from each section were analyzed.

### Staining of human biopsy specimen

Paraffin-embedded human hepatic tissues arrays (LV805a) from normal and patients with hepatic cirrhosis were purchased from US Biomax Inc. These slides were stained for human/mouse EphB2 and α-SMA as described above.

### Hydroxyproline assay

Liver tissues were homogenized and baked overnight at 105 °C in 12 N HCl. Hydroxyproline content was determined using a kit following the manufacturer’s instructions (BioVision).

### Plasma cytokines analysis

Cytokines analysis from plasma samples were performed using Singleplex Luminex^®^ kit for each analyte tested according to the manufacturer’s instructions (Invitrogen, ThermoFisher Scientific).

### Liver damage enzyme detection

Plasma samples from oil and CCl_4_-treated mice were processed in a single batch for determination of serum alanine aminotransferase (ALT) level using a DC Element chemistry analyser (HESKA).

### Western blot

Liver tissues and cells lysates were prepared with RIPA buffer containing 1× EDTA/proteinase-phosphatase inhibitor cocktail (Pierce). The lysate supernatant was stored at −80 °C until used for immunoblotting. Protein extracts were separated by SDS-PAGE electrophoresis and blotted onto nitrocellulose membrane. Blots were incubated overnight with the following primary detection antibodies: anti-α-SMA EPR5368, (1:2000, Abcam); anti-GAPDH GA1R and anti-β-actin (1:10000 ThermoFisher Scientific). The blots were then incubated with IRDye^®^ IgG secondary antibody conjugates (Li-Cor) and then revealed using an Odyssey^®^ gel documentation system following the manufacturer’s instruction (Li-Cor).

### Apoptosis assay

Apoptosis was evaluated in culture-activated HSCs and in frozen liver sections of mice chronically exposed to CCl_4_ using the *In Situ* Cell Death Detection Kit (Roche) following the manufacturer’s instruction. Images were captured using a confocal microscope and data analysed with ImageJ software.

### Cell proliferation assay

To assess HSCs proliferation, the BrdU assay was performed using the BrdU Cell Proliferation ELISA Kit (Abcam). A total of 1 × 10^4^ purified HSCs from EphB2*−/*− and WT littermates were plated in each well of a 96 well plate and culture for 6 days, with BrdU incorporated for the final 18 h of culture. The assay was performed following the manufacturer’s protocol.

### Statistical analysis

Statistical analyses were performed with the GraphPad Prism software 5.0 (Graph Pad software, USA) using the non-parametric Mann Whitney-U test. Values of *p* < 0.05 were considered statistically significant.

## Electronic supplementary material

Supplementary Information
