# Supplementary material for: EphB2 receptor tyrosine kinase promotes hepatic fibrogenesis in mice via activation of hepatic stellate cells
Source: Sci Rep. 2018 Feb 7;8:2532. doi: 10.1038/s41598-018-20926-9 (PMC5803231; doi:10.1038/s41598-018-20926-9)
Supplement: Supplementary file 1 — Supplementary Information [file 41598_2018_20926_MOESM1_ESM.pdf]

EphB2 receptor tyrosine kinase promotes hepatic fibrogenesis in mice via activation of hepatic stellate cells.

Patrice N. Mimche[1*], Choon M. Lee[2], Sylvie M. Mimche[2], Manoj Thapa[3], Arash Grakoui[3], Mark Henkemeyer[4], and Tracey J. Lamb[1]

[1]Division of Microbiology and Immunology, Department of Pathology, University of Utah School of Medicine, UT

[2]Department of Pharmacology, Emory University School of Medicine, Atlanta, GA

[3]Division of Medicine, Department of Infectious Diseases, Emory Vaccine Center, Emory University School of Medicine, Atlanta, GA

[4]Department of Neuroscience, University of Texas Southwestern Medical Center, Dallas, TX

***Correspondence and requests for materials should be addressed to P.N.M (email: patrice.mimche@path.utah.edu)**

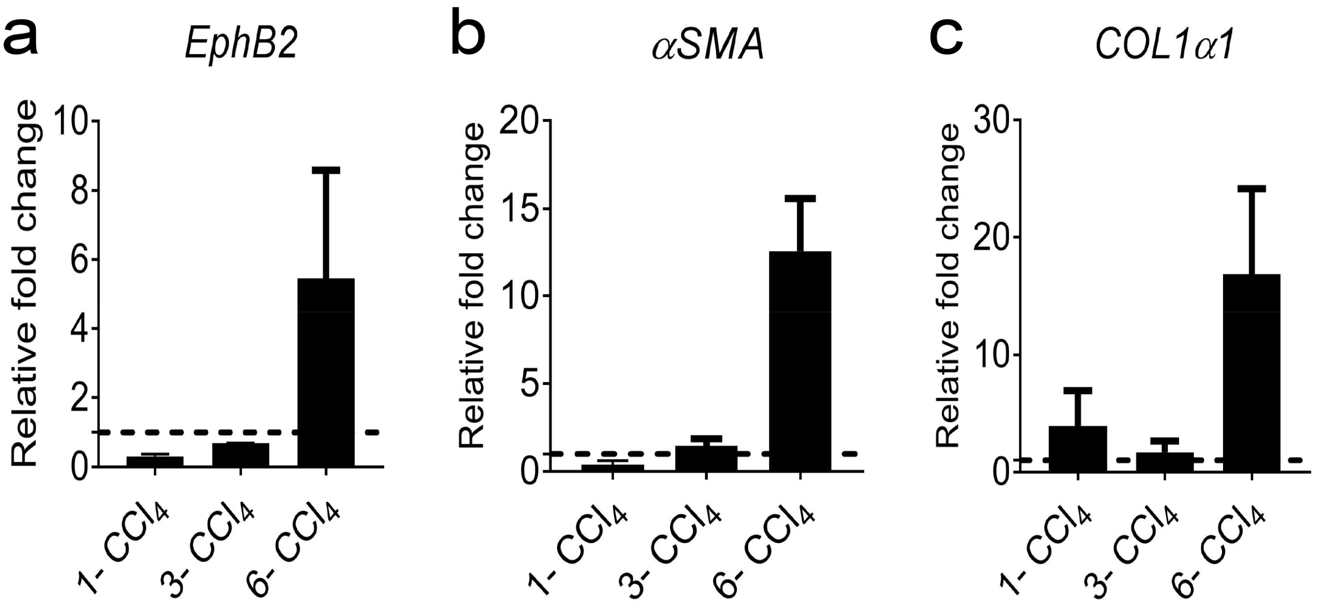

Figure S1: *EphB2, αSMA* and *COL1α1* mRNA levels in the liver after acute fibrogenic injury. (**a**) *EphB2,* (**b**) *αSMA* and (**c**) *COL1α1* mRNA levels were analyzed in livers of mice subjected to one dose (24-hour), three doses (three times a week) and six doses (three times a week for 2 weeks) of 10% CCl_4 or vehicle (oil) controls using RT-qPCR. Results are shown as fold change compared to vehicle-treated controls. Error bars represent mean ± SEM, n = 3 animals per group.

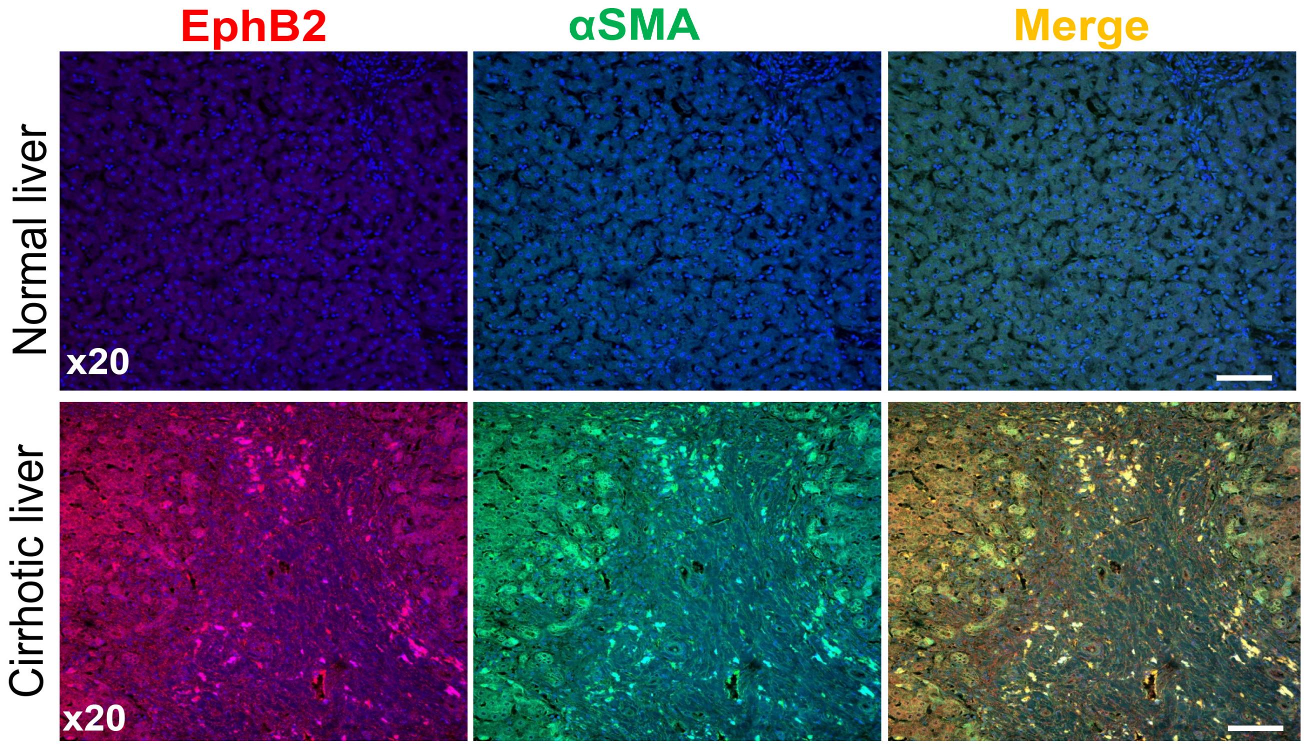

Figure S2: Expression of EphB2 increases in human liver cirrhosis.

FFPE liver sections of a normal subjects and a patients with liver cirrhosis (Unknown etiology, specimen obtained from a commercial source, Biomax Inc, US) were analyzed for expression of EphB2 protein by immunofluorescence confocal microscopy. Scale bar = 200μm.

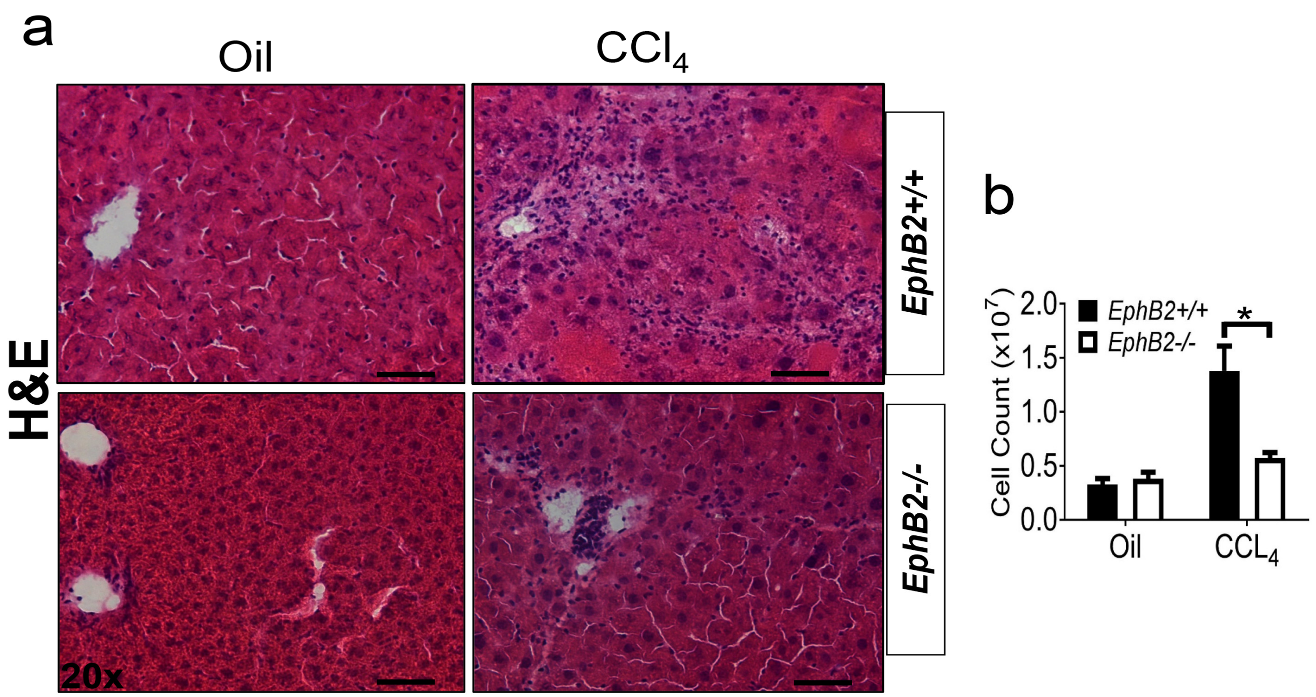

Figure S3: Evaluation of mononuclear cell infiltration in the liver of CCl_4-exposed mice. *EphB2-/-* mice and wild type littermates (WT) were chronically injected with CCl_4 twice a week for 6 weeks and sacrificed 72h after receiving the last dose of CCl_4.

(**a**) Representative microscopic images of Hematoxylin and Eosin staining depicting mononuclear infiltrates in the liver of *EphB2-/-* and littermates mice following chronic CCl_4 exposure. Scale bar = 200µm. (**b**) Enumeration of cellular infiltrates in the liver of *EphB2-/-* and littermates mice following chronic CCl_4 exposure. Data are mean ± SEM. n= 5 animals per group. * $p<0.05$.

Table S1: Mouse primer sequences.

Gene	Forward Primer	Reverse Primer
ephrin B1	TCGCAAGCATACACAGCAGCGG	ATGATGATGTCGCTGGGCTCGG
ephrin B2	CAGAAGAACCCTGCTTGCCTGG	AGCAAGCAGCCTTGACCTGC
ephrin B3	AGACTTTGGGGGAGTTGGTGCC	CAGCCCCGCAAAACCTAACAGC
EphB1	TTACAGCACAGGCCGAGGGGAGTTCG	AACTGGCCCATGATGCTCGCC
EphB2	ACGCCACGGCCATAAAAAGCCC	TTGCCACTGTAGCGCCCATAGC
EphB3	ATTGGGCATCAAGCCACCCAGC	TGCTCTGTAACCGAGGTGTCGC
EphB4	TTGAGCCCTGGGTGGCAATCCG	AGGCACCTCACGGTCAGTGG
EphB6	ACTCTAAGCTGCGAGCAGACGC	GCCAGGCTTGCCTTCTTGTCTGG
α-SMA	CGGGAGAAAATGACCCAGATT	AGGGACAGCACAGCCTGAATAG
Col1α1	GGAGAGTACTGGATCGACCCTAAC	ACACAGGTCTGACCTGTCTCCAT
PDGFRβ	CTCAGGGTTTTCCGCAATCA	TATCACCCCCTCCAGGAAGTC
TIMP-1	GCAGATATCCGGTACGCCTACA	TGCGGTTCTGGGACTTGTG
TGF-β1	ATCGACATGGAGCTGGTGAAA	TGGCGAGCCTTAGTTTGGA
TNF-α	TCTCATTCCTGCTTGTGGC	CACTTGGTGGTTTGCTACG
IL-6	ACACATGTTCTCTGGGAAATCGT	AAGTGCATCATCGTTGTTCATACA
IL-1β	GGGCTGCTTCCAAACCT	GATGTGCTGCTGCGAGA
CCR1	CCACTCCATGCCAAAAGACT	ATTAGGACATTGCCCACCAC
CCR2	GAAGGAGGGAGCAGTGTGTACAT	CCCCCACATAGGGATCATGA
CCR5	GGCCATGCAGGCAACAG	TCTCCAACAAAGGCATAGATGACA
CCL2	GGCTCAGCCAGATGCAGTTAA	CCTACTCATTGGGATCATCTTGCT
CXCL2	ACCAACCACCAGGCTACA	TCĜAGGGTCAAGGCAAACT
CXCL-10	GACGGTCCGCTGCAACTG	GCTTCCCTATGGCCCTCATT
HPRT	GGCCCACCTAGTCAGATAAGAGTTCC	ATGGCTCAGAAACGCTGCCGG

T-box21	CACCTGTTGTGGTCCAAGTTCA	CATTCGCCGTCCTTGCTTAG
GATA-3	CGAGACATAGAGAGCTACGCAATCT	CCTGAGTAGCAAGGAGCGTAGAG
GAPDH	TGTGTCCGTCGTGGATCTGA	TTGCTGTTGAAGTCGCAGGAG
